# In High-Light-Acclimated Coffee Plants the Metabolic Machinery Is Adjusted to Avoid Oxidative Stress Rather than to Benefit from Extra Light Enhancement in Photosynthetic Yield

**DOI:** 10.1371/journal.pone.0094862

**Published:** 2014-04-14

**Authors:** Samuel C. V. Martins, Wagner L. Araújo, Takayuki Tohge, Alisdair R. Fernie, Fábio M. DaMatta

**Affiliations:** 1 Departamento de Biologia Vegetal, Universidade Federal de Viçosa, Viçosa, Minas Gerais, Brazil; 2 Max-Planck-Partner Group at the Departamento de Biologia Vegetal, Universidade Federal de Viçosa, Viçosa, Minas Gerais, Brazil; 3 Max-Planck-Institut für Molekulare Pflanzenphysiologie, Potsdam-Golm, Germany; Tennessee State University, United States of America

## Abstract

Coffee (*Coffea arabica* L.) has been traditionally considered as shade-demanding, although it performs well without shade and even out-yields shaded coffee. Here we investigated how coffee plants adjust their metabolic machinery to varying light supply and whether these adjustments are supported by a reprogramming of the primary and secondary metabolism. We demonstrate that coffee plants are able to adjust its metabolic machinery to high light conditions through marked increases in its antioxidant capacity associated with enhanced consumption of reducing equivalents. Photorespiration and alternative pathways are suggested to be key players in reductant-consumption under high light conditions. We also demonstrate that both primary and secondary metabolism undergo extensive reprogramming under high light supply, including depression of the levels of intermediates of the tricarboxylic acid cycle that were accompanied by an up-regulation of a range of amino acids, sugars and sugar alcohols, polyamines and flavonoids such as kaempferol and quercetin derivatives. When taken together, the entire dataset is consistent with these metabolic alterations being primarily associated with oxidative stress avoidance rather than representing adjustments in order to facilitate the plants from utilizing the additional light to improve their photosynthetic performance.

## Introduction

Although light is the fundamental energy resource for plants since it fuels photosynthesis, thus driving plant growth, not only low but also high sunlight can limit plant performance. Whilst the shortages of key resources, such as light, can compromise growth and survival, plants conversely face heat, desiccation and excessive irradiance at high sunlight [1]. To cope with these stresses, plants have evolved a number of well-known biochemical, physiological and structural changes at the leaf and whole-plant levels that enable them to adjust to a particular set of light conditions [2], [3]. In any case, given that plants perform photosynthesis and assimilatory processes in a continuously changing environment, energy production in the various cell compartments and energy consumption in endergonic processes have to be well adjusted to the prevailing conditions [4]. In this regard, homeostasis is crucial in maintenance of all cellular functions, one means by which this is achieved is by ensuring that the pools of ATP/ADP, NAD(P)H/NAD(P) and other redox carriers remain at balanced ratios [4]–[6].

Recent years have witnessed a widespread and steady adoption of post genomic tools in an attempt to better understand the cellular response to a broad range of stresses including amongst others carbon starvation [7], [8], nutritional stress [9], [10], light stress [11], oxidative stress [12] and UV-B stress [13]. Over the past few years, metabolomics has been established as a powerful tool to characterize the complex metabolic response of plants to a range of biotic and environmental stresses [14], [15]. The advantage of metabolomics is that it provides a functional measure of cellular status and ultimately in its broadest form even describes an organism phenotype [16]. Noticeably fewer studies have, however, been performed, to date, that set out to phenotype the metabolic adjustments occurring in concert with photosynthetic responses to varying light supply. Furthermore, most metabolomics-based studies dealing with carbon economy have focused on model woody temperate species such as poplar (e.g., [17]) and particularly on herbaceous plants such as Arabidopsis (e.g., [10], [11]), tobacco (e.g., [12]) and tomato (e.g., [9], [18]), whereas almost no studies focus on tropical woody species.

Coffee is one of the most important commodities in the international agricultural trade, generating over 90 billion dollars each year, with approximately 500 million people involved in its processing, from cultivation to final consumption [19]. It is a tropical perennial shrub evolved in the understory of the African forest and is traditionally considered to be a shade-demanding species. In early plantations, coffee bushes were planted under taller shade trees to simulate their natural habitat. However, in many situations modern coffee cultivars can grow well and even produce greater yields in the sun than in the shade; therefore, shading has been abandoned as a regular cultural practice in several regions worldwide [19], [20]. The species displays low rates of net carbon assimilation (*A*), with maximum values typically ranging from 4 to 11 µmol m^−2^ s^−1^ with current atmospheric CO_2_ concentration and saturating light [19]. The combination of low *A* of coffee leaves with high light often results in linear electron fluxes that can be more than several times greater than required for the observed *A* [21]. Given that photoinhibition and photodamage have not usually been observed in coffee even in leaves highly exposed to full sunlight [21]–[25], other pathways for photosynthetic electron flow, such as photorespiration and the Mehler-peroxidase reaction pathway (the PS I-mediated photoreduction of O_2_), have been suggested as key dissipators of the excess energy in coffee leaves under such conditions [21], [23], [24]. The coffee shrub additionally exhibits high concentrations of secondary metabolic compounds which are both C-rich *e.g*., phenols and N-rich *e.g*., purine alkaloids, and the total pools of these compounds increase markedly with increasing light supply [26]. Compounds such as phenolics constitute one of the most abundant classes of plant secondary metabolites and are believed to act as screening agents against harmful UV radiation and potential scavengers of reactive oxygen species (ROS) [27], [28]. Furthermore, diversion of C skeletons from the primary metabolism to the expensive synthesis of secondary metabolites may account for as much as 30% of the C flow in plants [29], which may help to avoid photosynthetic down-regulation and photoinhibition [26]. Ultimately, all of these adjustments to cope with high light would be anticipated to result in a complex metabolic reprogramming; however, this has yet to be experimentally investigated in coffee.

Given the facts described above, we hypothesize that major metabolic adjustments are required to allow maintenance of successful performance in coffee plants grown under full sunlight conditions. Our main goal was to investigate how the metabolic machinery of coffee adjusts itself to varying light supply and whether these adjustments are supported by clear reprogramming of primary and secondary metabolism. To attain this goal, we compared coffee plants grown under low light or high light conditions by analyzing photosynthetic traits, antioxidant capacity, pyridine and adenylate nucleotide pool sizes as well as both primary and secondary metabolite profiles. Our combined results demonstrate a clear metabolic reprogramming allowing the plant to cope with HL and specifically identified a greater abundance of metabolites likely involved in protection against light-induced oxidative stress. We discuss these data in the context of metabolic adjustments acting not to improve photosynthetic performance *per se* but rather to afford protection associated with energy dissipation and ROS scavenging.

## Material and Methods

### Plant material, growth conditions and experimental design

The experiment was conducted in Viçosa (20°45′S, 42°54′W, 650 m in altitude) in south-eastern Brazil. Uniform seedlings of *Coffea arabica* L. cv ‘Catuaí Vermelho IAC 44’ obtained from seeds were grown in 12 L pots containing a mixture of soil, sand and composted manure (4∶1∶1, v/v/v). Plants were grown either under full (100%) sunlight conditions (HL, high light) or under low light (10% full sunlight) in a shade environment (LL, low light). The shade enclosure was constructed using neutral-density black nylon netting, and the plants were kept in these conditions for 12 months before measurements. Throughout the experiment, the plants were grown under naturally fluctuating conditions of temperature and air relative humidity, and were fertilized and irrigated as necessary. The pots were randomized periodically to minimize any variation within each light environment. For all samplings and measurements, the youngest, fully expanded leaves, corresponding to the third or fourth pair from the apex of plagiotropic branches, were used. For biochemical analyses, leaf discs were collected in cloudless days at about midday, prior to flash freezing in liquid nitrogen and subsequent storage at −80°C until analysis.

The experiment was arranged in a completely randomized design. The means presented in Tables and Figures were obtained from six independent replications per treatment of single plant experimental plots (one plant per pot).

### Photosynthetic gas exchange

The net CO_2_ assimilation rate (*A*), stomatal conductance (*g*_s_) and the internal CO_2_ concentration (*C*_i_) were determined simultaneously with conducting measurements of chlorophyll *a* fluorescence using a portable open-flow gas exchange system (LI-6400XT, LI-COR, Lincoln, NE, USA) equipped with an integrated fluorescence chamber head (LI-6400-40, LI-COR Inc.). Measurements were conducted on attached leaves from 08:00 to 09:00 hours (solar time), which is when *A* was at its maximum, at 40 Pa CO_2_ partial pressure under artificial photosynthetic photon flux density (PPFD), *i.e*., 200 or 1,000 µmol photons m^−2^ s ^−1^ at the leaf level (for LL and HL leaves, respectively). These PPFD intensities approximately corresponded to the ambient irradiance that was intercepted by the sampled leaves (in their natural angles) for each light treatment. All measurements were performed at 25°C, and the leaf-to-air vapour pressure deficit was maintained at *c*. 1.0–1.5 kPa, while the amount of blue light was set to 10% of PPFD in order to maximize the stomatal aperture.

Photosynthetic light-response curves (*A*/PPFD) were produced by decreasing the PPFD in 12 steps from 1500 to 0 µmol m^−2^ s^−1^ at 25°C. The leaf tissues were initially exposed to a 5-Pa CO_2_ partial pressure for 5 min to allow stomatal opening; the *A*/PPFD curves were subsequently obtained at 40 Pa of CO_2_ partial pressure. The light saturation point (LSP) was determined from these curves. Further details are presented elsewhere [25].

The responses of *A* to *C*_i_ were determined at 1,000 µmol photons m^−2^ s^−1^, at 25°C, under ambient O_2_ following the protocol described in Long and Bernacchi [30]. Measurements started at 40 Pa CO_2_ and once a steady-state was achieved the CO_2_ partial pressure was gradually lowered to 5 Pa and then increased stepwise up to 200 Pa. The maximum rate of carboxylation (*V*_cmax_) and maximum rate of carboxylation limited by electron transport (*J*_max_) were then estimated by fitting the mechanistic model of CO_2_ assimilation using the Excel spreadsheet provided by Sharkey et al. [31]; the required kinetic parameters of Rubisco for running this model were determined for coffee as reported by Martins et al. [32]. Corrections for the leakage of CO_2_ and water vapor into and out of the leaf chamber of the Li-6400–40 have been applied to all gas-exchange data, as described by Rodeghiero et al. [33].

### Chlorophyll *a* fluorescence parameters

Previously dark-adapted (30 min) leaf tissues were illuminated with weak modulated measuring beams (0.03 µmol m^−2^ s^−1^) to obtain the initial fluorescence (*F*_0_). Saturating white light pulses of 8,000 µmol photons m^−2^ s^−1^ were applied for 0.8 s to ensure for maximum fluorescence emissions (*F*_m_), from which the variable-to-maximum chlorophyll fluorescence ratio, 
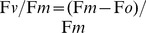
, was calculated.

In light-adapted leaves, the actual PSII photochemical efficiency (*Φ*_PSII_) was determined by measuring steady-state fluorescence (*F*_s_) and maximum fluorescence during a light-saturating pulse of *c*. 8,000 µmol m^−2^ s^−1^ (*F*_m_'), following the procedures of Genty et al. [34]:
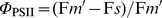
After the light-saturating pulse, the leaf was transiently darkened and and the minimal fluorescence after induction was determined (*F*_0_') using weak far-red light in order to fully oxidize the PSII acceptor side. The non-photochemical quenching coefficient (NPQ) was calculated according to Logan et al. [35] as:
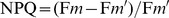



### Estimation of electron flow devoted to photosynthesis and photorespiration

The partitioning of electrons between photosynthesis (*J*_c_) and photorespiration (*J*_o_) was obtained as described elsewhere [30], [36] using the values of electron transport rate (ETR), *A* and mitochondrial respiration rate in the light (*R*_d_), as follows:
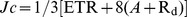





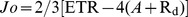



In order to improve the accuracy of *J*_c_ and *J*_o_ estimation, the ETR was calibrated to account for uncertainties in α (leaf absorptance) and β (PSII optical cross section) [32]. The calibration was performed under non-photorespiratory conditions in an atmosphere containing less than 1 kPa O_2_ using the linear plot of *Φ*_PSII_ and *Φ*_CO2_, based on Valentini et al. [36]:
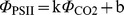





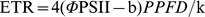
where 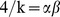, *k* and b were obtained through the linear fit of *Φ*_PSII_ versus *Φ*_CO2_. The *k* and b values were 9.1±0.13 and −0.008±0.0028 under LL, and 8.3±0.24 and −0.014±0.0055 under HL.

The *R*_d_ was determined according to the ‘Laisk-method’ [37], using the *y* axis intersection of *A*/*C*_i_ (internal CO_2_ concentration) curves performed at three different PPFD intensities (750, 250 and 75 µmol m^−2^ s^−1^).

### Antioxidant activity and cellular damage

The total antioxidant capacity was estimated via the ferric reducing antioxidant power assay of Benzie and Strain [38]. In this assay, antioxidants are used as reductants in a redox-linked colorimetric technique, employing an easily reduced oxidant system present in stoichiometric excess.

Cellular damage was analyzed based on malondialdehyde accumulation, estimated as the content of total 2-thiobarbituric acid-reactive substances, as detailed in Lima et al. [39].

### Adenylate, uridinylate and pyridine nucleotide analyses

Metabolite extraction was performed by grinding the lyophilized materials with liquid nitrogen and immediate addition of the appropriate extraction buffers. The levels of NAD(H) and NADP(H) were determined as described previously by Schippers et al. [40]. For adenylate and uridinylate analyses, trichloroacetic acid extracts were prepared according to Jelitto et al. [41] and were measured by high performance liquid chromatography (HPLC), using a Bio-Tek HPLC system (pump-system 522, HPLC autosampler 560, HPLC 535 UV detector) with a Partisil-SAX anion exchange column (10 µm; Knauer) with ammonium dihydrogen phosphate buffers (Merck) exactly as described previously [42]. Data analysis was performed with chromeleon 6.80 software (Dionex).

### Metabolite profiling

To obtain a broad overview of the major pathways of primary metabolism an established GC-MS-based metabolite profiling method [43] was used to quantify the relative metabolite levels in response to the imposed treatments. Metabolite analysis was performed using approximately 75 mg of fully expanded dry leaf tissues. The extraction, derivatization, standard addition, and sample injection were performed exactly as described previously [44]. The GC-MS system was comprised of a CTC CombiPAL autosampler, an Agilent 6890N gas chromatograph and a LECO Pegasus III TOF-MS running in EI+ mode. Chromatograms and mass spectra were evaluated by using Chroma TOF 1.0 (Leco, http://www.leco.com/) and TagFinder 4.0 software [45]. Metabolite identification was manually supervised using the mass spectral and retention index collection of the Golm Metabolome Database in comparison to database entries of authentic standards [46], [47]. Peak heights of the mass fragments were normalized on the basis of the dry weight of the sample and the added amount of an internal standard (ribitol). Data are normalized with respect to the mean response calculated for the LL (to allow statistical assessment, individual plants from this set were normalized in the same way). Data presentation and experimental details follow recent recommendations [48] and are recorded in Data Set S1.

To study secondary metabolite profiles, LC-MS analysis was conducted as described previously [49]. Metabolites were identified and annotated based on comparisons with data in our previous publications [50], [51] with co-elution profile of Arabidopsis leaf and tomato pericarp extracts, metabolite databases [52], [53] and standard compounds (3-chlorogenic acid and quercetin-3-*O*-Glc-6″-*O*-Rha). The peaks of other phenolic compounds including feruloylquinic acid derivatives, dicaffeoylquinic acid derivatives and mangiferin were annotated by following the spectrum data found previously in coffee leaf and bean (fruit) extracts [54]–[56].

### Additional assays

Starch pools were quantified in lyophilized leaf samples, as described in detail elsewhere [57]. Total nitrogen and nitrate were measured in oven-dried leaf tissues (72 h at 70°C) as described previously [58].

### Statistical analyses

Data are expressed as the means ± standard error. Student's *t*-tests were used to compare the parameters between treatments. All statistical analyses were carried out using Microsoft Excel.

## Results

### Photosynthesis and energy dissipation

The maximum PPFD intercepted by LL leaves was *c*. 200 µmol photons m^−2^ s^−1^, against more than 1,000 µmol photons m^−2^ s^−1^. We assessed the actual *A* under the prevailing PPFD conditions in either treatment, thus more properly reflecting the differences in actual CO_2_ fixation rate between treatments. On an area basis, the HL plants displayed higher *A* (193%) and *g*_s_ values (122%) than those of LL plants whereas *C*_i_ did not differ significantly in response to the light treatments (Table 1). The potential photosynthetic capacity (assessed under saturating PPFD) was also higher in HL than in LL plants, as inferred from the higher (*c*. 56%) values of both *V*_cmax_ and *J*_max_ (Table 1). However, due to adjustments in specific leaf area (Table 2), the differences on a per mass basis in the values of these traits were much narrower: *A* was 60% higher in HL than in LL plants whereas *V*_cmax_ and *J*_max_ did not differ significantly between these plants (Table 1).

**Table 1:** The net CO_2_ assimilation rate (*A*), maximum rate of carboxylation (*V*_cmax_), maximum rate of carboxylation limited by electron transport (*J*_max_), light saturation point (LSP), oxygenative (*J*_o_) and carboxylative (*J*_c_) electron flows as well as the *J*_o_ */J*_c_ ratio and mitochondrial respiration rate in the light (*R*_d_) of coffee plants grown under low or high light (10 or 100% full sunlight, respectively).

Parameters	Treatments			
	Area basis		Mass basis	
	Low light	High light	Low light	High light
*A*	4.0±0.21	11.7±0.60**	0.10±0.01	0.16±0.01**
*g*_s_	60±7	133±17**	–	–
*C*_i_	276±13	241±7	–	–
*V*_cmax_	26.8±1.64	41.7±2.37**	0.61±0.04	0.58±0.03 ^ns^
*J*_max_	71.1±4.36	110.7±3.39**	1.6±0.10	1.6±0.05 ^ns^
LSP	340±20	607±34**	–	–
*J*_c_	20.3±0.53	60.3±3.60**	0.46±0.01	0.84±0.05**
*J*_o_	6.4±0.61	26.7±2.62**	0.15±0.01	0.37±0.04**
*J*_o_ */J*_c_	0.31±0.02	0.44±0.02**	–	–
*R*_d_	0.12±0.02	0.28±0.08*	0.003±0.000	0.004±0.001 ^ns^

*A*, *V*_cmax_, and *R*_d_ are expressed on area (µmol CO_2_ m^−2^ s^−1^) or mass basis (µmol CO_2_ g^−1^ DW s^−1^); *J*_max_, *J*_c_ and *J*_o_ are expressed on area (µmol electrons m^−2^ s^−1^) or mass basis (µmol electrons g^−1^ DW s^−1^); LSP is expressed on area (µmol photons m^−2^ s^−1^) basis. *A*, *J*_c_ and *J*_o_ were measured or estimated for the prevailing light conditions and under ambient CO_2_ in each treatment; *V*_cmax_ and *J*_max_ were estimated using *A*/*C*_i_ curves under saturating light. *n* = 6± SE. Significance: ^ns^ not significant, *P<0.05, **P<0.01.

**Table 2:** The specific leaf area (SLA), total nitrogen (N_total_), nitrate (NO_3_^−^) and starch concentrations of coffee plants grown under low or high light (100 or 10% full sunlight, respectively).

Parameters	Treatments	
	Low light	High light
SLA (m^2^ kg^−1^)	22.9±4.5	14.0±2.7**
N_total_ (g kg^−1^ DW)	39.9±1.5	34.8±1.4*
NO_3_^−^ (g kg^−1^ DW)	1.4±0.11	0.3±0.06**
Starch (g kg^−1^ DW)	20.7±2.2	29.2±4.9*

*n* = 6± SE. Significance: **P<0.01, *P<0.05.

Given that HL plants received levels of PPFD well above their light saturation point (607 µmol photons m^−2^ s^−1^; Table 1), adjustments in light use and dissipation are required to avoid an excess energy that would otherwise lead to photoinhibition. Indeed, we found that the NPQ was higher (283%) in HL than in LL plants (Table 3), suggesting an enhanced energy dissipation as heat. Despite this excess energy, the *F*_v_/*F*_m_, as measured at midday, was at or above 0.80 (Table 3), which can be interpreted as a complete lack of photoinhibition of the photosynthetic apparatus [31]. We next measured the total antioxidant capacity using the ferric reducing antioxidant power assay (Table 3). We observed a 5.5 fold increase in total antioxidant capacity in HL leaves in comparison to their LL counterparts; such enhanced antioxidant potential may well have helped the HL plants to maintain high *F*_v_/*F*_m_ values despite the adversity of the light conditions. Importantly, irrespective of differences in the capacities for photosynthesis and antioxidant defenses, the concentration of malondialdehyde, which can be used as a marker for oxidative stress, was essentially the same regardless of the light supply (Table 3). Collectively, these data strongly indicate that the HL plants did not suffer from oxidative stress.

**Table 3:** The variable-to-maximum chlorophyll fluorescence ratio (*F*_v_/*F*_m_), non-photochemical quenching coefficient (NPQ), total antioxidant capacity and malondialdehyde concentration of coffee plants grown under low or high light (10 or 100% full sunlight, respectively).

Parameters	Treatments	
	Low light	High light
*F*_v_/*F*_m_	0.82±0.02	0.80±0.01^ns^
NPQ	0.58±0.01	2.22±0.26**
Antioxidant capacity (mmol Fe^2+^ g^−1^ FW)	104±9	672±40**
Malondialdehyde (nmol g^−1^ FW)	71.3±3.6	74.2±2.0^ns^

*n* = 6± SE. Significance: ^ns^ not significant, **P<0.01.

To gain insights into the relative importance of the photorespiratory pathway as a mechanism for dissipating excess energy, we estimated the oxygenative and carboxylative electron flows as well as the *J*_o_ */J*_c_ ratio (Table 1) under the prevailing growth PPFD and ambient CO_2_ in either treatment. Regardless of whether expressed on area or mass basis, *J*_c_ and *J*_o_ were higher in HL than in LL plants. Moreover, the *J*_o_ */J*_c_ ratio was estimated to be significantly higher in HL than in LL plants (Table 1), suggesting that higher dissipation of excess energy occurred through the photorespiratory pathway under HL conditions leading to a changed balance between RuBP oxygenation and carboxylation.

### Nucleotide pool sizes

**Figure 1:**
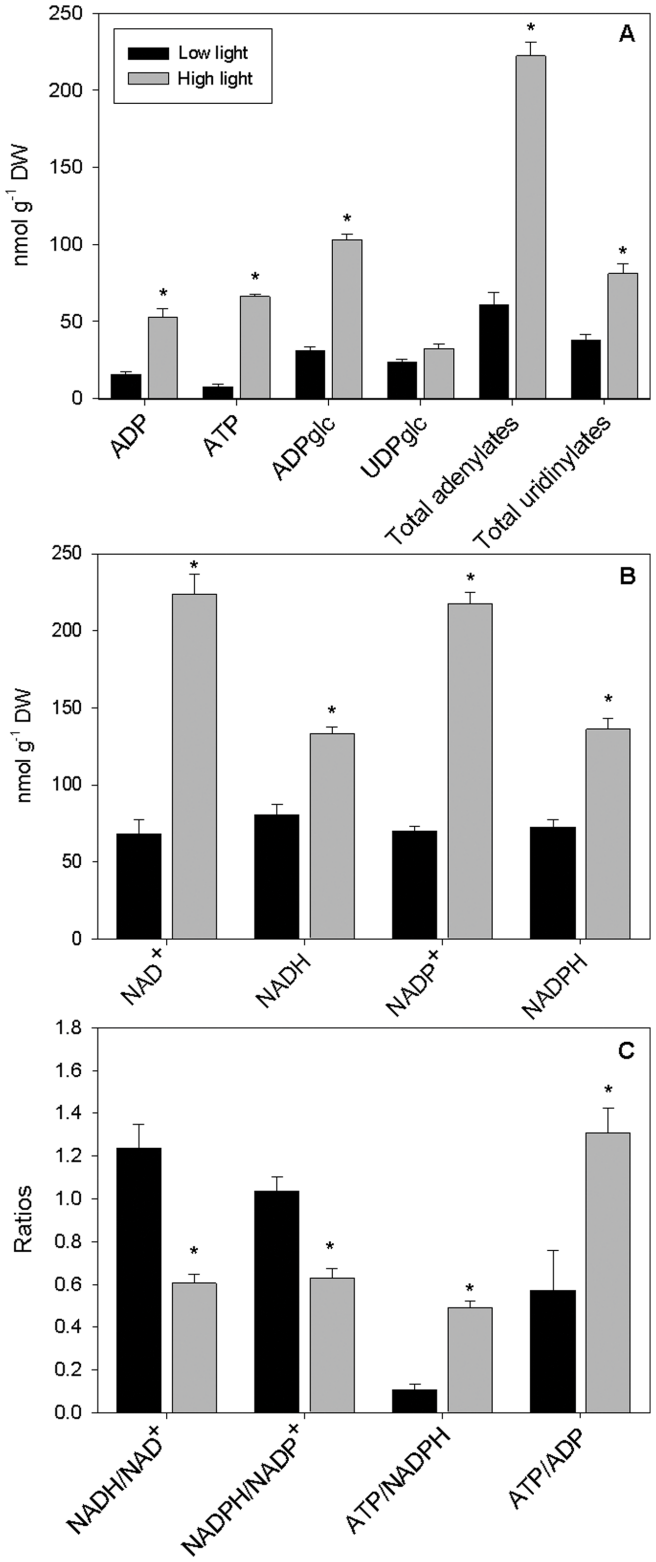
The pyridine and adenylate nucleotide pool sizes. The concentrations of adenylates, uridinylates (A) and pyridine nucleotides (B), and some ratios (C), of coffee plants grown under low or high light (10 or 100% full sunlight, respectively). *n* = 6± SE. The means for high-light plants marked with an asterisk differ significantly from those for low-light plants (*P*<0.05).

Overall, large differences in the pools of adenylates, uridinylates and pyridine nucleotides were noticeable between LL and HL plants (Fig. 1A,B). Compared to LL plants, their HL counterparts displayed larger concentrations (on a dry weight basis) of total adenylates (265%), total uridinylates (108%), ADP-glucose (175%), the sum of NADH and NAD^+^ (140%), NADPH and NADP^+^ (148%) and the sum of ATP and ADP (409%), whereas UDP-glucose levels did not differ significantly in response to the light treatments (Fig. 1A,B). The ATP/ADP ratio was 1.3 in HL against 0.6 in LL plants (Fig. 1C). Intriguingly, the NADH/NAD^+^ and NADPH/NADP^+^ ratios were approximately 0.6 in HL plants and 1.2–1.0 in LL individuals (Fig. 1C). The ATP/NADPH ratio ranged from 0.11 in LL plants to 0.49 in their HL relatives (Fig. 1C), indicating that NADPH is produced in excess with respect to ATP, especially in LL plants.

### Primary and secondary metabolite profiling

**Figure 2:**
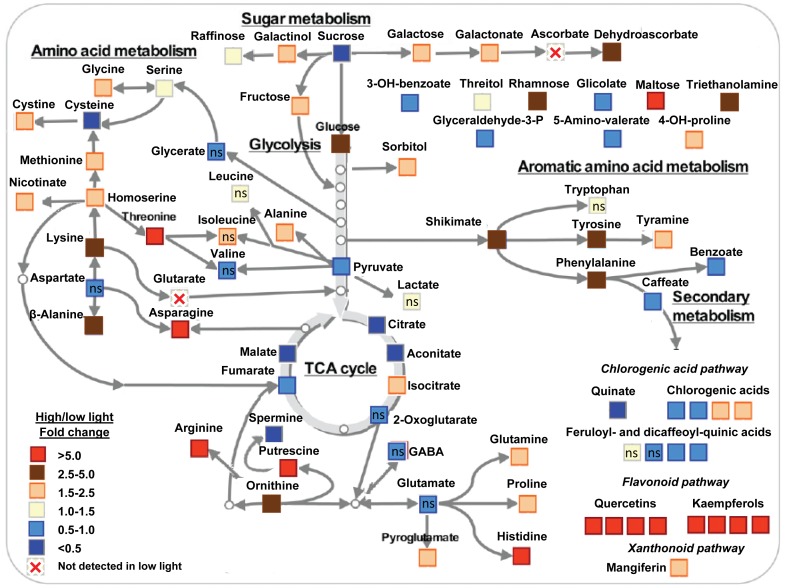
The major metabolic alterations of coffee plants in response to the light treatments. These alterations, as observed in plants grown under low or high light (10 or 100% full sunlight, respectively), are synthesized in a schematic summary wherein they are mapped onto metabolic pathways. The colors indicate the proportional content of each putatively identified metabolite among the samples, as determined by the average peak response. For the whole metabolite profiling, only 12 metabolites were identified as not significantly affected by the light treatments (marked as ‘ns’). Metabolites were determined as described in “Materials and Methods”. *n* = 6± SE.

Among the 63 successfully annotated compounds related to the primary metabolism, considerable differences in the levels of a wide range of amino acids, organic acids, sugars, sugar alcohols and polyamines were evident. Of these compounds, 63% increased in their content under HL conditions relative to LL conditions. To provide an easy overview, the major metabolic changes observed were synthesized in a schematic summary wherein they are mapped onto metabolic pathways (Fig. 2). The full data set is additionally presented in Table 4. A total of 24 amino acids were determined. With the exception of alanine, leucine, valine, isoloeucine, glutamate, aspartate and tryptophan, whose concentrations did not differ significantly between HL and LL plants, and cysteine, which was 55% less abundant in HL than in LL plants, all of the remaining amino acids were more abundant in HL plants than in their LL counterparts. Notably, compared with LL plants, HL plants displayed an overall higher concentration of amino acids that was coupled with a modestly lower total N concentration (13%) and a markedly lower nitrate concentration (78%) (Table 2). Taken together, these data indicate that N metabolism is likely to be constrained by low light availability.

**Table 4:** The relative primary metabolite profile of coffee plants.

	LL	HL			LL	HL
**Amino acids**						
Alanine	1±0.07	2.1±0.09**		Isoleucine	1±0.19	1.7±0.25^ ns^
β-Alanine	1±0.13	2.5±0.18**		Leucine	1±0.08	1.3±0.13^ ns^
Arginine	1±0.16	5.2±0.90**		Lysine	1±0.24	3.5±0.35**
Asparagine	1±0.06	5.0±0.19**		Methionine	1±0.02	1.7±0.13**
Aspartate	1±0.05	0.9±0.03^ ns^		Phenylalanine	1±0.11	3.8±0.33**
Cysteine	1±0.05	0.5±0.03**		Proline	1±0.12	2.4±0.17**
Cystine	1±0.18	2.1±0.17**		4-OH-Proline	1±0.07	1.6±0.13**
Glutamate	1±0.12	0.8±0.08^ ns^		Serine	1±0.10	1.3±0.08*
Glutamine	1±0.09	2.1±0.16**		Threonine	1±0.07	5.8±0.46**
Glycine	1±0.13	1.6±0.12*		Tryptophan	1±0.26	1.3±0.33^ ns^
Histidine	1±0.16	10.9±0.57**		Tyrosine	1±0.14	4.4±1.00*
Homoserine	1±0.08	2.3±0.17**		Valine	1±0.03	1.0±0.06^ ns^
**Organic acids**						
Aconitate	1±0.03	0.2±0.01**		Glycerate	1±0.12	0.8±0.01^ ns^
Ascorbate	ND			Glycolate	1±0.06	0.7±0.03**
Benzoate	1±0.04	0.5±0.01**		Isocitrate	1±0.05	2.2±0.23**
3-OH-Benzoate	1±0.08	0.8±0.06*		Lactate	1±0.14	1.2±0.17^ ns^
GABA	1±0.13	0.8±0.05^ ns^		Malate	1±0.04	0.5±0.04**
Caffeate	1±0.03	0.7±0.02**		Nicotinate	1±0.06	1.9±0.07**
Citrate	1±0.17	0.2±0.03**		Pyroglutamate	1±0.07	1.8±0.09**
Dehydroascorbate	1±0.07	3.2±0.08**		Pyruvate	1±0.04	0.7±0.04**
Fumarate	1±0.04	0.5±0.06**		Quinate	1±0.02	0.5±0.04**
Galactonate	1±0.06	1.9±0.16**		3-Caffeoyl-quinate	1±0.03	1.2±0.08^ ns^
Glutarate	ND			Shikimate	1±0.17	2.8±0.24**
2-Oxo-Glutarate	1±0.12	0.8±0.03^ ns^		5-Amino-Valerate	1±0.02	0.6±0.04**
**Sugars**						
Fructose	1±0.14	2.1±0.20**		Raffinose	1±0.09	1.3±0.12**
Galactose	1±0.09	1.7±0.05**		Rhamnose	1±0.01	3.0±0.15**
Glucose	1±0.18	2.6±0.18**		Sucrose	1±0.02	0.5±0.04**
Maltose	1±0.13	5.7±0.28**				
**Sugar alcohols**						
Galactinol	1±0.02	2.5±0.04**		Threitol	1±0.08	1.4±0.15*
Sorbitol	1±0.02	1.8±0.02**				
**Polyamines**						
Ornithine	1±0.13	4.3±1.06*		Threonine	1±0.07	5.8±0.46*
Putrescine	1±0.09	6.6±0.29**		Triethanolamine	1±0.10	4.8±1.39**
Spermine	1±0.02	0.4±0.02**		Tyramine	1±0.09	1.7±0.01**

The plants were grown under low light (LL) or high light (HL) (10 or 100% full sunlight, respectively). Results for LL plants were set as unit. Data are normalized with respect to the mean response calculated for the LL (to allow statistical assessment, individual plants from this set were normalized in the same way). ND  =  metabolites not detected in the LL treatment. Significance: ^ns^ not significant, * *P*<0.05, **P<0.01

One key organic acid associated with the glycolytic pathway and five organic acids directly involved in the tricarboxylic acid (TCA) cycle were identified as significantly affected by the light treatments: pyruvate, malate and fumarate, and particularly citrate and aconitate, were more abundant in LL plants than in HL plants, whereas the opposite was true for isocitrate (Fig. 2, Table 4). 2-oxoglutarate pools were similar between LL and HL individuals. We successfully identified a further 18 organic acids. Of particular interest is the observation that shikimate pools were about two fold higher in HL plants and these elevated levels were accompanied by higher tyrosine and phenylalanine pools whereas glutarate and ascorbate were only present at detectable levels in the HL samples.

With the exception of sucrose pools, which were 55% lower in HL plants than in LL plants, the monosaccharides glucose, fructose, galactose and rhamnose, oligosaccharides such as maltose and raffinose, as well as polyols such as threitol, sorbitol and galactinol, all were more abundant in HL plants than in their LL counterparts (Fig. 2, Table 4). We also identified six polyamines and, with the exception of spermine, all were at remarkably higher levels in HL plants (Fig. 2, Table 4).

Next, we performed the determination of secondary metabolite pools by LC-MS. Of the total peaks detected, we identified and annotated eight compounds as hydroxycinnamic acids, eight as flavonols and one xanthonoid compound putatively identified as mangiferin (Fig. 2; Table 5). Whereas no clear pattern was found for variations in hydroxycinnamic acids when comparing HL and LL plants, we identified a significant increase in the levels of mangiferin and a dramatic increase (up to more than 100 fold) in the levels of four kaempferol and four quercetin derivatives including rutin in HL plants relative to LL individuals.

**Table 5:** The relative secondary metabolite profile of coffee plants.

	LL	HL
**Phenolic acids**		
3-CGA	1±0.07	0.6±0.05**
5-CGA	1±0.08	1.7±0.11**
putative CGA related	1±0.06	0.7±0.06**
4-CGA	1±0.10	1.8±0.10**
putative FQA	1±0.13	1.2±0.04^ns^
putative 3,4-diCQA	1±0.10	0.5±0.08**
putative 3,5-diCQA	1±0.11	0.9±0.13^ns^
putative 4,5-diCQA	1±0.06	0.7±0.09*
**Flavonols**		
Quercetin-3Glc-Hex-DeHex	1±0.24	32.9±1.18**
Quercetin-3Glc-Hex	1±0.14	13.0±0.89**
Rutin (quercetin-3Glc-6″Rha)	1±0.10	103.8±10.36**
Quercetin-3Glc	1±0.09	22.4±2.47**
Kaempferol-3Glc-Hex-DeHex	1±0.30	27.4±2.17**
Kaempferol-3Glc-Hex	1±0.12	5.5±0.52**
Kaempferol-3Glc-6″Rha	1±0.07	141.0±37.29*
Kaempferol-3Glc	1±0.43	23.5±5.70*
**Xanthonoid**		
putative Mangiferin	1±0.11	2.3±0.18**

The plants were grown under low light (LL) or high light (HL) (10 or 100% full sunlight, respectively). Results for LL plants were set as unit. Data are normalized with respect to the mean response calculated for the LL (to allow statistical assessment, individual plants from this set were normalized in the same way). Chlorogenic acid (3-CGA) and Rutin have been identified by standard compounds. Flavonol-3Glcs were annotated by co-elution profile of Arabidopsis leaf and tomato fruit extracts (Rohrmann *et al*. 2011; Wu *et al*. 2012). The other phenolics such as cryptochlorogenic acid (4-CGA), neochlorogenic acid (5-CGA), feruloylquinic acids (FQA), dicaffeoylquinic acids (diCQA) and mangiferin were annotated based on comparison of the tables of coffee profile (Mondolot *et al*. 2006; Alonso-Salces *et al*. 2009; Campa *et al*. 2012) Significance: ^ns^ not significant, *P<0.05, **P<0.01

## Discussion

### The capacity of the photosynthetic machinery is similar between LL and HL plants

We first demonstrated that proportionally larger differences for the combined pools of NADPH and NADP^+^ (which essentially reflects photosynthetic activity [59]) and adenylates between HL and LL plants took place (Fig. 1A,B) relative to the difference in actual *A* on a per mass basis between these individuals (Table 1). Furthermore, we also demonstrated that the photosynthetic capacity (assessed through *V*_cmax_ and *J*_max_) on a mass basis of LL and HL plants is similar (Table 1). It is therefore tempting to assume the pyridine and adenylate nucleotide pool sizes are in excess and, hence, the biochemical capacity of the photosynthetic machinery of HL individuals is most likely greater than necessary to support their actual photosynthetic rates.

### Enhanced consumption of reductant equivalents seems to play a major role for avoiding oxidative stress

Regardless of the light supply, we found a relative excess of NADPH over ATP (Fig. 1C), suggesting that reductants are unlikely to limit *A* under the conditions of this study. Intriguingly, we observed that the NADP system was more oxidized under HL conditions (Fig. 1B), in sharp contrast with other studies (e.g., [59], [60]). This is an unexpected result given that the electron pressure within the photosynthetic electron transport chain must be greater at HL than at LL supply. In any case, our results suggest that HL individuals should use large amounts of NADPH in metabolic pathways other than carbon fixation. Rapid oxidation of NADPH may be an effective way of avoiding harmful over-reduction and inactivation of photosynthetic electron transport chain and thus ROS formation [6], [61]. In addition, a higher rate of NADP^+^ reduction may lead to increasing thylakoid energization, which, in turn, would be anticipated to enhance energy dissipation associated with increased transthylakoidal proton gradient [62], [63] and thus provide more energy for ATP synthesis [5], [64]. These hypotheses are additionally consistent with the higher NPQ (Table 3) and ATP/ADP ratio (Fig. 1C) of the HL plants. Notably, these plants also displayed relatively lower NAD(P)H/NAD(P)^+^ ratio (Fig. 1C), which may be linked to an up-regulation of the glycine decarboxylase complex and hence photorespiration [65]. Collectively, these results suggest that an orchestrated metabolic reprogramming to enhance reducing equivalent consumption occurs in HL plants.

All of the above described responses concerning changes in cellular redox status are consistent with enhanced activities of the photorespiratory and the Mehler-peroxidase pathways (for a review see Miyake [63]) in HL plants. Of course, other alternative pathways including the operation of the mitochondrial alternative oxidase and the cyclic flow of electrons may also act as energy escape valves [4], [63]. Notably, higher cyclic electron flow decreases the generation of NADPH while it simultaneously increases ATP production [64], which is consistent with higher ATP/NADPH ratios observed in HL plants (Fig. 1). Importantly, regardless of mechanisms for dissipating the excess energy, we demonstrated that coffee plant is able to remarkably increase its total antioxidant capacity (through a range of enzymatic and non-enzymatic antioxidants [22]–[24], [66] with no apparent light-induced symptoms of oxidative stress as illustrated by the comparable malondialdehyde levels and high *F*_v_/*F*_m_ values of LL and HL plants (Table 3).

### Both primary and secondary metabolism are reprogrammed in response to changing light availability

Given that the availability of UDP-glucose, a key intermediate of the sucrose biosynthetic pathway, was similar in both LL in HL individuals (Fig. 1A), and no apparent up-regulation of sucrose consumption was evident in LL plants (Fig. 2), we contend that the surplus of sucrose in LL individuals, coupled with decreases in starch (29%) pools (Table 2), was chiefly a consequence of decreased carbohydrate requirements and/or decreased ability for sucrose export. Elevated pool sizes of sucrose, pyruvate and several intermediates of the TCA cycle would suggest, at a first glance, that central metabolism was not constrained by any noticeable decrease in carbon availability in LL plants. Nevertheless, reduced levels of intermediates of glycolysis and TCA cycle in HL plants that correlated with increases in a range of amino acids are also observed (Fig. 2, Table 4). This response contrasts with the results found in high-light-grown Arabidopsis [10], [67] which displayed lower levels of several amino acids under these conditions. Returning to the coffee data presented here it should be stressed that many amino acids that accumulate in HL plants, concomitantly with accumulation of sugar alcohols and polyamines, have been shown to be involved in multiple stress responses [16].

In HL plants many compounds potentially involved in several known paths for protection against stress were up-regulated (Fig. 2, Table 4). First, sugars and polyols such as raffinose, galactinol and sorbitol, and amino acids such as proline, which have been revealed to protect plants not only from osmotic stress but also from various other stresses. It has been proposed that this protection is at least in part due to their ROS scavenging properties [68], [69], which is consistent with the fact that oxidative stress is a common feature of the majority of both biotic and abiotic stresses [70]. Secondly, glycine, serine and glutamine, which are directly involved in or closely associated with photorespiratory metabolism. Thirdly, â-alanine which is involved in CoA biosynthesis, which, in turn, is an essential co-factor not only in primary metabolism but also in secondary metabolism including the phenylpropanoid pathway [71]. Fourthly, several polyamines which appear to be an integral component of plant response to stress through as yet unresolved mechanisms [72]. Fifthly, shikimate pools and up-regulated concentrations of aromatic amino acids, which can serve as precursors of hydroxycinnamic acids, alkaloids, lignins and flavonoids [73] and thereby potentially also linked to the increases in â-alanine. Finally, ascorbate which is fundamental for proper operation of the Mehler-peroxidase pathway [6] as well as in recycling flavonoid radicals to their reduced forms [74]. From the above, we suggest that all of these metabolic reprogramming that occurred in HL plants could be described in general terms as a stress response. Alternatively, given that many of the amino acids whose pools were largely up-regulated (e.g., threonine, asparagine, arginine, proline) are synthesized through pathways that are energetically expensive [75], it is conceivable that amino acid biosynthesis induced by high light is part of a strategy to balance the redox status of the plant, thus ultimately contributing to avoid ROS formation and photooxidative damages.

In contrast to hydroxycinnamates such as chlorogenic acids, which responded only slightly to HL conditions, flavonol pools were dramatically up-regulated (Fig. 2, Table 5), confirming previous studies suggesting a major role of these compounds in the response mechanisms to excess light [74]. We showed that HL induced similar responses in terms of accumulation of both orthodihydroxy B-ring-substituted flavonoids such as quercetin derivatives and their monohydroxy B-ring-substituted counterparts such as kaempferol derivatives, in contrast to other studies that reported a preponderance of accumulation of quercetin over kaempferol derivatives [76]–[78], which are supposed to be less effective in ROS scavenging than quercetin derivatives [79]. Irrespective of this difference, both kaempferol and quercetin derivatives appear to have a greater ability to inhibit the generation of ROS as compared with other phenylpropanoids [78], and both can be synthesized in chloroplasts [79], [80]. Moreover, the synthesis of these flavonols is more expensive than that of simpler phenylpropanoids in terms of reduced carbon, reducing equivalents and ATP costs [81]; hence the light-induced up-regulation of flavonols might be an interesting way to divert excess energy towards compounds with additional antioxidant activities [79]. In addition, we observed an up-regulation of mangiferin levels in HL plants, a xanthonoid that is also believed to play a function in ROS scavenging [56]. From the above, we propose that reprogramming of the metabolic machinery towards increased synthesis of phenolics such as flavonols and mangiferin, as well as amino acids and the expensive synthesis of purine alkaloids such as caffeine [26], might represent important energy escape valves as a fundamental part of the underlying general mechanism by which coffee plants cope with high light levels.

## Conclusions

We demonstrated that the coffee plant was successfully able to adjust its metabolic machinery to HL conditions through marked increases in its antioxidant capacity associated with large consumption of reductant equivalents. We also demonstrated that both primary and secondary metabolism undergo extensive reprogramming under HL conditions. This can be expected given the sessile nature of plant growth coupled with the survival in a constantly changing environment requires plant metabolism to operate in an extremely dynamic manner. Apparently, metabolic alterations took place chiefly to avoid oxidative stress rather than representing adjustments for the HL plants to benefit from the extra light to improve their photosynthetic performance. We suggest that for species such as coffee, which evolved in shade habitats but can successfully grow under full sunlight conditions, large energy expenditure through metabolic reprogramming may be crucial for improved growth and fitness when light energy is so plentiful. In this context, interactions of the photosynthetic machinery with the metabolic activities in both mitochondria and chloroplast are seemingly essential for the proper maintenance of intracellular redox gradients to allow for considerable rates of energy dissipation and efficient photosynthesis. Confirmation and identification of the exact point of control as well as the signaling mechanism behind such response and indeed of the precise biological function of light availability in the regulation of metabolism remain as important questions that should be addressed in future studies. Relatively inexpensive high-throughput sequencing technology in combination with emerging metabolomics technologies [82], [83] will likely facilitate the mining of yet more genes and metabolites involved in this specialized response.

## Supporting Information

Data Set S1 **Overview and checklist features of the metabolite reporting list.**
(XLS)
